# A rare case with synchronous gastric gastrointestinal stromal tumor, pancreatic neuroendocrine tumor, and uterine leiomyoma

**DOI:** 10.1186/s12957-016-1051-x

**Published:** 2016-11-15

**Authors:** Elena Arabadzhieva, Atanas Yonkov, Sasho Bonev, Dimitar Bulanov, Ivanka Taneva, Alexandrina Vlahova, Tihomir Dikov, Violeta Dimitrova

**Affiliations:** 1 Department of General and Hepato-pancreatic Surgery, University Hospital “Alexandrovska”–Sofia, 1 Georgi Sofiiski Str, 1431 Sofia, Bulgaria; 2 Medical University–Sofia, 15 Acad. I. E. Geshov Bul, 1431 Sofia, Bulgaria; 3 Department of General and Clinical Pathology, 2 Zdrave Str, 1431 Sofia, Bulgaria

**Keywords:** Pancreatic neuroendocrine tumor, Gastrointestinal stromal tumor, Uterine leiomyoma, Chromogranin A, Synaptophysin

## Abstract

**Background:**

Although gastrointestinal stromal tumors (GISTs) are the most common mesenchymal tumors of the gastrointestinal tract, they comprise less than 1% of all gastrointestinal tumors. Neuroendocrine tumors (NET) of the gastro-enteropancreatic system are also rare, representing about 2% of all gastrointestinal neoplasms. Pancreatic localization of NET is extremely uncommon—these tumors are only 1–5% of all pancreatic cancers.

**Case presentation:**

We describe an unusual case with triple tumor localization—a gastric tumor, a formation in the pancreas, which involves the retroperitoneal space, and a uterine leiomyoma. The exact diagnosis was confirmed with immunohistochemical study after surgical treatment of the patient. Distal pancreatic resection, splenectomy, partial gastrectomy, omentectomy, and hysterectomy were performed. The histological examination proved an epithelioid type of gastric GIST. Immunostaining showed focal positive expression of c-kit and no mitotic figures per 50 HPF. Histology of the pancreatic and retroperitoneal formation proved a well-differentiated NET with origin from the islets of Langerhans. The immunohistochemical study demonstrated co-expression of chromogranin A and synaptophysin.

**Conclusions:**

This is the fourth case published so far of a patient with synchronous pancreatic NET and gastric GIST. The main objective of the study is to present a unique case because we have not found any reports for coexistence of the described three types of neoplasm, as in our patient, and we hope that it will be valuable in the future investigations about the genesis, diagnosis, and treatment of these types of tumors.

## Background

Although gastrointestinal stromal tumors (GISTs) are the most common mesenchymal tumors of the gastrointestinal (GI) tract, they comprise less than 1% of all GI tumors [1]. Their annual incidence is 11–19.6 cases per 100,000 individuals [2, 3]. Neuroendocrine tumors (NETs) of the gastro-enteropancreatic (GEP) system are also rare, usually sporadic, representing about 2% of all GI tumors [4]. Pancreatic localization of NETs is extremely uncommon—these neoplasms are only 1–5% of all the pancreatic cancers and their incidence does not exceed five to one million [4]. On the other hand, uterine fibroids (also known as leiomyomas or myomas) are the commonest benign uterine tumors associated with significant morbidity to nearly 40% of women during their reproductive years and sometimes even after menopause [5]. Because the coexistence of the described three tumors is quite unusual and unique, we present the details of our case. We used the collected data about the patient from the medical records in our hospital and from the available medical documentation of her previous hospital stays and medical treatment.

## Case presentation

A 60-year-old female was admitted with symptoms of weakness and single occurrence of black and tarry stools. The patient’s co-morbidities included arterial hypertension and diabetes. A uterine myoma had been diagnosed a few years ago. Familial disease history included a mother with arterial hypertension and diabetes, died of heart attack, and a father, died of a stroke. Her aunt died of a gastric cancer.

The physical examination revealed that the abdomen was respiratory movable, without palpable pain, but with two palpable formations with dense texture. The first formation was movable and localized in the epigastrium, measuring about 7 cm. Under the umbilical horizontal, there was an immobile tumor with smooth surface and about 25 cm in size. The rectal digital examination did not establish presence of melena.

**Fig. 1:**
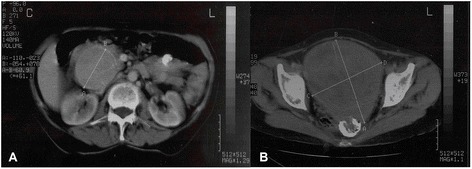
**a** CT image of retroperitoneal tumor located under the liver. **b** CT image of pelvic formation

The ultrasound revealed a soft tissue formation with heterogeneous structure, located in epigastric region and infiltrating the liver. Endoscopic examination showed a small duodenal ulcerative lesion. Because of this, abdominal computed tomography (CT) was performed. It demonstrated a heterodense formation, localized in the retroperitoneal space, under the liver, without infiltration of it. The tumor was about 70 mm and had smooth and sharp outlines (Fig. 1a). There was another formation in the pelvis with similar features, but 143/124 mm in size. The uterus was behind it with suspected infiltration of the organ (Fig. 1b). The results from routine laboratory tests were within normal limits. The exact diagnosis was confirmed with immunohistochemical study after surgical treatment of the patient.

The intraoperative exploration revealed an enlarged uterus, involved by a huge fibroid, measuring more than 20 cm. There was a tumor formation, involving the front gastric wall in the pyloric area, which was with irregular shape, dense texture, and diameter about 6–7 cm. A second tumor, measuring about 7 cm, was detected in the retroperitoneal space. The formation was connected to another tumor in the pancreatic body, which was about 5 cm in size. The tumors were with solid consistency and oval shape and did not involve other organs and tissues.

Splenectomy and distal pancreatic resection was performed, and the retroperitoneal tumor was carefully extirpated. Because of the presence of gastric tumor, a Billroth’s operation II and omentectomy were carried out. The surgical procedure ended with hysterectomy, considering the large uterine myoma.

**Fig. 2:**
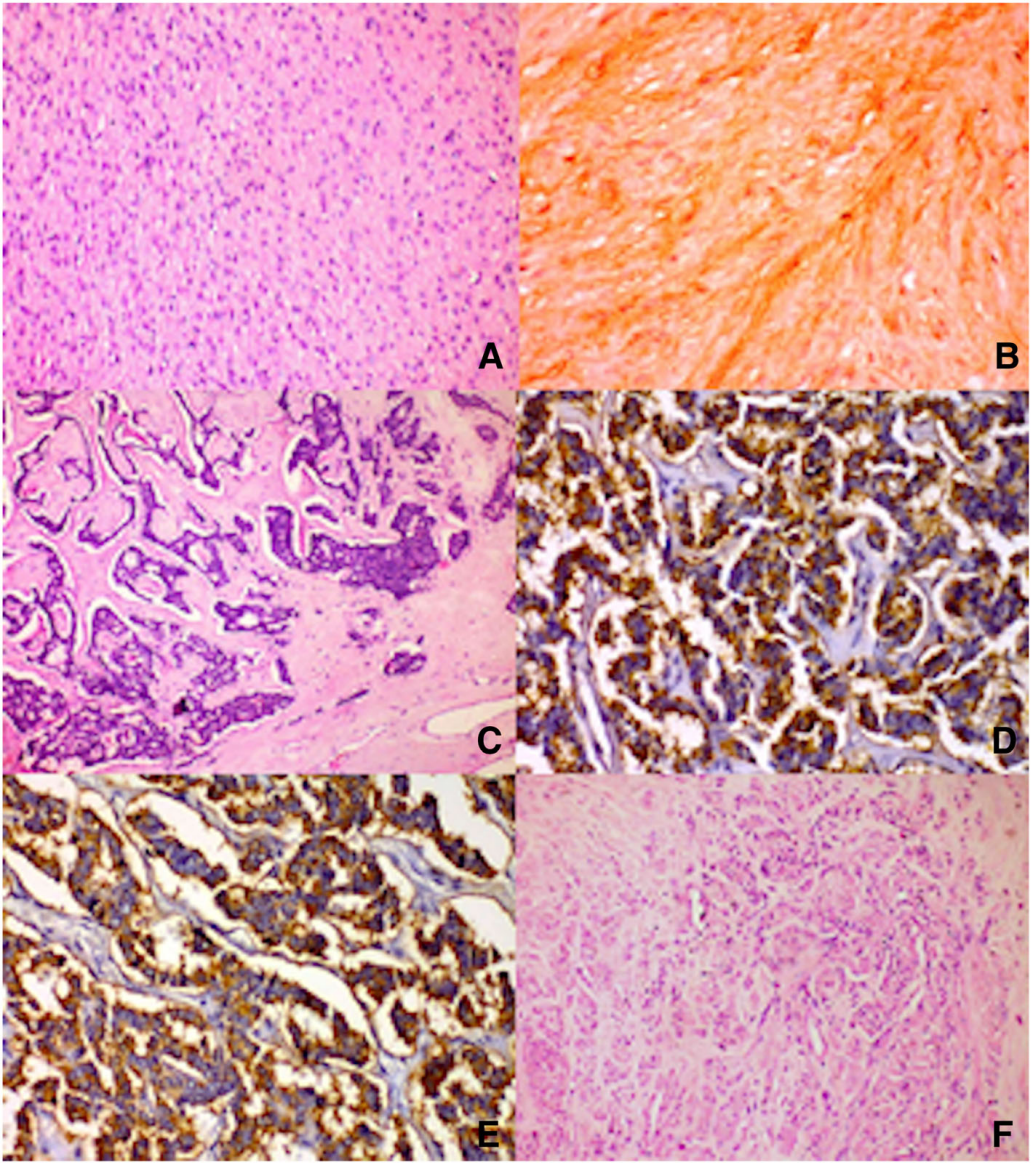
Histology. **a** Microscopic section of the gastric GIST (H&E ×200). **b** Immunohistochemical reaction for c-kit (×400). **c** Microscopic section of the pancreatic NET (H&E ×100). **d** Immunohistochemical reaction for chromogranin A (×400). **e** Immunohistochemical reaction for synaptophysin (×400). **f** Microscopic section of the leiomyoma of the uterus (H&E ×200)

The gross pathological evaluation revealed a well-demarcated gastric tumor, just beneath a hyperemic mucosa, extending through the gastric wall to the grossly unremarkable serous. Cut surfice showed central cavity. The histological examination proved an epithelioid type of gastric GIST. Immunostaining showed focal positive expression of c-kit (CD117). No mitotic figures were found per 50 HPF (10 mm^2^), subsequently confirmed by immunohistochemical examination with Ki-67 (Fig. 2a and b). The patient has refused the investigation of the GIST mutational status KIT/PDGFRA.

The retroperitoneal tumor was firm and encapsulated, with cut surface, variegated with small cystic and hemorrhagic areas. Histology proved a well-differentiated NET with origin from the islets of Langerhans. Immunohistochemical tumor cells demonstrated co-expression of chromogranin A and synaptophysin. Tumor proliferative activity, as estimated by Ki-67, was low—there was intensive nuclear expression in 1 to 2% of tumor cells (Fig. 2c, d, e).

Specimen from the pancreas contained a well-delineated firm tumor with heterogenous cut surface and unremarkable spleen. Microscopically, tumor tissue was proved identical to the retroperitoneal one.

Lastly, microscopic examination of the signifiacantly enlarged uterus found a leiomyoma without further qualifiers (Fig. 2f).

Postoperative period was uneventful. Octreoscan showed no evidence of metastatic focus, and because of the low risk of progression of GIST, according to Miettinen’s classification, the patient did not carry out any additional treatment. A regular follow-up was performed. Six years after the operation, the patient was in good health without clinical, ultrasound, and CT imaging data for the recurrent disease.

The present study involved a literature search for relevant articles using Pubmed and Web of Science. To minimize the chance of missing an important study, a manual search of the references of all articles found in our search was also performed. The keywords were as follows: “neuroendocrine tumors,” “leiomyoma,” and “gastrointestinal stromal tumors.” GISTs are rare, usually sporadic neoplasms arising from, or differentiating along, a line similar to the interstitial cells of Cajal [1]. These neoplasms most commonly occur in the stomach (60–70%), followed by the small intestine (20–30%), duodenum (4–5%), rectum (4–5%), colon (<2%), and esophagus(<1%) [1, 6]. Gastric GISTs can be incidentally detected or usually manifested with non-specific symptoms, such as nausea, vomiting, and abdominal pain or, most often, with bleeding, [4] as in our case. Pathologic diagnosis is based on both unique microscopic features (fusiform, epithelioid or mixed type) and immunohistochemical techniques (CD-117, CD34, actin, desmin, S-100, and Ki-67) with counting of the number of mitoses per 50 HPF [1, 7, 8]. Different types of mutations can be found in KIT and PDGFRA genes encoding a receptor tyrosine kinases type III (RTC). Risk stratification of GISTs according to Miettinen et al., Fletcher’s classification and UICC classification is based on tumor size, mitotic index, and localization of the tumor [1, 6–8]. Achieved resection margins (R0 or R1) and capsule rupture are other prognostic factors [8]. The standard treatment of GISTs is surgical resection [1, 7, 8]. Targeted medical therapy by tyrosine kinase inhibitors is recommended only for GIST that is marginally resectable or resectable with a risk of significant morbidity [4, 7, 9].

Pancreatic NETs are also uncommon neoplasms which originate from diffuse neuroendocrine cells [4]. Pancreatic NETs may be divided into functional (F-pancreatic NET) and non-functional (NF-pancreatic NET), depending on their ability to secrete biologically active hormones [10]. Because of this, as in our case, the NF-pancreatic NETs can be asymptomatic before they reach a significant tumor size when the symptoms are result from the mass effect and involvement of the adjacent organs [10]. Therefore, diagnosis of NF-pancreatic NETs depends on detailed biochemical, radiographic, and pathologic examination [10]. The histopathological and immunohistochemical (chromogranin A and synaptophysin) examinations play an essential role in establishing the diagnosis and prognosis of a neuroendocrine tumor [11]. By WHO classification, G1 tumors have a variable structure, either with insular, trabecular, acinar, diffuse or mixed patterns, and by a monomorphic cytology with low atypia and rare if any mitosis (<2/10 HPF), Ki-67 <2% [4, 10]. G2 tumors show focal moderate cytological atypia with a few scattered mitotic figures (2–20/10 HPF), Ki-67 3-20%, and spotty necrosis [4, 10]. G3 tumors demonstrate a solid growth pattern; the tumor cells are small, round or oat cell-like with marked nuclear pleomorphism, brisk mitotic activity (>20/10 HPF), Ki-67 >20%, and sizeable areas of tumor necrosis [4, 10]. In our case, the pancreatic NET was determined as G1.

GISTs have been described to coexist with other neoplasms, the percentage of such cases ranging from 4.5 to 33% [4]. They frequently develop as a result of hereditary diseases, such as neurofibromatosis type 1 (NF1), Carney’s triad (GIST, paraganglioma and pulmonary chondroma), Carney’s dyad (paraganglioma and gastric GIST), and familial GIST [3]. On the other hand, GEP NETs may be related to increased risk of synchronous malignancies [4]. That can be the result from the fact that 10% of NETs will be associated with an inherited genetic syndromes, such as MEN1 (hyperparathyroidism, pancreatic NETs in up to 75% of cases, and pituitary tumors), Von Hippel-Lindau disease (NF-pancreatic NETs) in 10–20% of patients, cystadenomas, hemangioblastomas and adenocarcinoma, tuberous sclerosis, and NF1 [10]. The coexistence of gastric GIST and pancreatic NETs is extremely a rare condition and there are only three more cases, described in the literature, excluding our case [4, 12, 13]. Although there are very few cases of NF1-related concurrent GEP NETs and GISTs, there is no evidence that the mutation in the NF1 involved in the pathogenesis of GIST is the same mutation existing in NF1 patients with coexistent GISTs and GEP NETs [12]. In the described four cases (with our patient), there was also no family history or clinical findings suggestive of NF-1, so mutations in non-NF1 patients with coexistent GISTs and GEP NETs are still unclear. Interestingly, in the three cases with synchronous gastric GIST and pancreatic NETs, as in our case, the grade of GIST is low or very low, and the pancreatic NETs are non-functional in three of the four cases [4, 12, 13].

Although uterine leiomyomas are the commonest benign uterine tumors [5], the coexistence with pancreatic NETs and gastric GIST is quite unusual and we believe that our patient is the first described case with such a tumor combination. Pathogenesis of uterine leiomyomas is not clearly known, too. Mechanotransduction, the response of cells to the mechanical forces such as compression and stretch and steroid-dependent growth are some of the mechanisms of occurrence of leiomyomas [5, 14]. There are some studies that revealed genetic alterations in patients with uterine leiomyomas—a chromosomal rearrangement of 12q14-15 reflecting the rearrangement of the HMGA2 allele and a mutation of mediator complex subunit 12 (MED12), a transcription factor gene [14, 15]. However, not all patients with leiomyomas display these genetic alterations [14, 15], and there are no evidence that these mutations have relation to the occurrence of pancreatic NETs and GIST. The limitations of our study are related to the patient’s refusal for performance of genetic investigations, so we could not prove a specific genetic disorder that may provoke the concomitant appearance of these neoplasms. However, we hope the presentation of this case could be useful in future studies about the genesis, diagnosis, and treatment of these types of tumors and correlations between them.

## Conclusions

We describe an unusual case with triple tumor localization—gastric GIST, pancreatic NET, and uterine leiomyoma. We have not found any reports for coexistence of the described three types of tumors, which makes our case interesting, unique, and valuable.

## Abbreviations

CT: Computed tomography
F-pancreatic NET: Functional pancreatic NET
GEP: Gastro-enteropancreatic
GI: Gastrointestinal
GIST: Gastrointestinal stromal tumor
NET: Neuroendocrine tumor
NF-pancreatic NET: Non-functional pancreatic NET
NF1: Neurofibromatosis type 1
